# Identification and validation of an anoikis-associated gene signature to predict clinical character, stemness, IDH mutation, and immune filtration in glioblastoma

**DOI:** 10.3389/fimmu.2022.939523

**Published:** 2022-08-25

**Authors:** Zhongzheng Sun, Yongquan Zhao, Yan Wei, Xuan Ding, Chenyang Tan, Chengwei Wang

**Affiliations:** ^1^ Department of Neurosurgery, The Second Hospital of Shandong University, Jinan, China; ^2^ Department of Neurosurgery, Dongying City District People’s Hospital, Dongying, China; ^3^ Department of Neurology, The Second Hospital of Shandong University, Jinan, China

**Keywords:** glioblastoma, anoikis, tumor microenvironment, stemness index, immunotherapy, pan-cancer analysis

## Abstract

**Background:**

Glioblastoma (GBM) is the most prominent and aggressive primary brain tumor in adults. Anoikis is a specific form of programmed cell death that plays a key role in tumor invasion and metastasis. The presence of anti-anoikis factors is associated with tumor aggressiveness and drug resistance.

**Methods:**

The non-negative matrix factorization algorithm was used for effective dimension reduction for integrated datasets. Differences in the tumor microenvironment (TME), stemness indices, and clinical characteristics between the two clusters were analyzed. Difference analysis, weighted gene coexpression network analysis (WGCNA), univariate Cox regression, and least absolute shrinkage and selection operator regression were leveraged to screen prognosis-related genes and construct a risk score model. Immunohistochemistry was performed to evaluate the expression of representative genes in clinical specimens. The relationship between the risk score and the TME, stemness, clinical traits, and immunotherapy response was assessed in GBM and pancancer.

**Results:**

Two definite clusters were identified on the basis of anoikis-related gene expression. Patients with GBM assigned to C1 were characterized by shortened overall survival, higher suppressive immune infiltration levels, and lower stemness indices. We further constructed a risk scoring model to quantify the regulatory patterns of anoikis-related genes. The higher risk score group was characterized by a poor prognosis, the infiltration of suppressive immune cells and a differentiated phenotype, whereas the lower risk score group exhibited the opposite effects. In addition, patients in the lower risk score group exhibited a higher frequency of isocitrate dehydrogenase (IDH) mutations and a more sensitive response to immunotherapy. Drug sensitivity analysis was performed, revealing that the higher risk group may benefit more from drugs targeting the PI3K/mTOR signaling pathway.

**Conclusion:**

We revealed potential relationships between anoikis-related genes and clinical features, TME, stemness, IDH mutation, and immunotherapy and elucidated their therapeutic value.

## Introduction

Glioblastoma (GBM), which is classified as a grade IV glioma, is the most prominent primary brain tumor in adults (1). The median survival of patients with GBM is approximately 12.6 months with a 5-year survival rate of less than 10% (2). The highly aggressive nature of GBM makes it impossible to completely remove by surgery, leading to its high recurrence rate and treatment failure (3, 4).

Cell‒cell adherence and interaction with the extracellular matrix (ECM) are implicated in several mandatory cellular processes, including migration and proliferation (5). Anoikis is due to the rupture of cell‒cell or cell-ECM attachment, leading to this specific form of programmed apoptosis, which helps maintain tissue homeostasis by eliminating misplaced or dislodged cells (6). The triggering of anoikis occurs mainly through the interaction of two apoptotic pathways, i.e., interference with mitochondria or activation of cell surface death receptors (7, 8). Anoikis was first described in epithelial and endothelial cells and was found to be an important mechanism of cancer invasion and metastasis (9). The onset of anoikis resistance can help detached cells circumvent death signaling pathways, allowing cells to survive under unfavorable conditions (10, 11). Numerous studies have found that PDK4 upregulation is directly implicated in the acquisition of chemoresistance in lung cancer and promotes tumor cell proliferation *in vivo* and *in vitro* (12). In addition, it was confirmed that the Nm23-ITGA5 pathway plays a key role in breast cancer cell invasion, and regulation of this pathway could potentially be utilized to prevent the establishment of breast cancer cell metastasis (13). However, few studies have systematically evaluated the implications of anoikis in GBM, although these anoikis genes play a non-negligible role in tumorigenesis tumor invasion and tumor infiltration.

Immunotherapy is of great interest in cancer-related treatment, which fights tumors by boosting the patient’s immune system. Under normal circumstances, it is now widely accepted that immune cells in the tumor microenvironment (TME) can distinguish and eradicate cancer cells, which is referred to as immunosurveillance (12, 14). However, cancer cells can modulate the host immune system to evade immune surveillance by recruiting immunosuppressive cell populations and downregulating tumor immunogenicity (15, 16). Stemness is used to evaluate the similarity of tumor cells to stem cells (17). This feature is mainly assessed using the mRNA expression–based stemness index (mRNAsi) as well as the epigenetic regulation-based index (EREG-mRNAsi). Stemness indices range from 0 to 1, where a value of 0 means low similarity to stem cells, whereas a value of 1 indicates high similarity to stem cells (18–20). Malta et al. found that tumor development was related to the progressive loss of the differentiated phenotype and the acquisition of progenitor-like, stem cell characteristics (21). Undifferentiated tumors of primary origin are more likely to undergo aggressive migration or form distant metastasis, ultimately leading to tumor progression (22, 23). It is essential to explore the alteration of the tumor microenvironment and infiltration of immune cells for immunotherapy and stemness index-based studies.

The classification of central nervous system tumors marks a shift in tumor diagnosis by incorporating molecular and phenotypic features into the tumor classification, thereby narrowing the defined subgroups (24). On the basis of genetic transcription characteristics, GBM can be divided into five subtypes [mesenchymal (MES), classical (CL), proneural (PN), neural, and proliferative]. Among others, the EMT facilitates radiochemotherapy resistance (25, 26). MES subtype patients exhibit aggressive and poor prognosis, whereas PN subtype patients exhibit a better prognosis (27–29). Different subtypes of GBM have different epigenomic markers. Several epigenomic markers have also shown prognostic and/or predictive values. Further typing of GBM will help to understand the differences between subtypes of GBM, which is important for developing more personalized and accurate treatment plans for GBM.

In this research, we first explored the differential expression of anoikis-related genes in GBM and potential subtypes in GBM. Second, the association between the differential expression of anoikis-related genes and the TME as well as different GBM subtypes was analyzed. Then, we constructed a risk score model based on the differential expression of anoikis-related genes to predict patient prognosis as well as response to immunotherapy. Finally, pancancer analysis further validated the reasonableness and credibility of the risk score model. Exploring anoikis-associated gene expression patterns not only expands our understanding of the aggressiveness of GBM but also contributes to the development of more personalized and precise therapy strategies.

## Materials and methods

### Data collection

The RNA-seq transcriptome data and clinical information [including gender, age, subtype, IDH status, survival, and CpG island methylator phenotype (CIMP) status] of GBM were downloaded from The Cancer Genome Atlas (TCGA)-GBM (https://portal.gdc.cancer.gov/) and GlioVis [GlioVis - Visualization Tools for Glioma Datasets (cnio.es)]. Somatic mutation counts and copy number variation (CNV) also were downloaded from the TCGA database. Twenty-seven anoikis-related genes were acquired from the gene set enrichment analysis (GSEA) (http://www.gsea-msigdb.org/gsea/index.jsp).

### Characteristics of the anoikis-related genes

First, we researched the interaction relationship between different anoikis-related genes. Meanwhile, the somatic mutation prevalence, the genetic locus, and CNV of anoikis-related genes were analyzed. We also analyzed the profile of 27 anoikis-related genes expression in different subtypes of GBM (including MES, CL, and PN). Merging the GlioVis datasets, we performed a univariate Cox regression analysis of 27 anoikis-related genes and forest plots were drawn.

### Immunohistochemistry

We collected section from paraffin-embedded tissues of human glioma and peritumor. We dewaxed and dissociated the sections and rehydrated sections. After heating in tris-EDTA buffer, we blocked slides using 5% gout serum and incubated slides with primary antibody (PTK2, 1:800, #3285; Cell Signaling Technology) (ITGA5, 1:100, #ab150361; Abcam) at 4°C overnight. Then, the slides were incubated with secondary antibody, and the images were captured using a Leica DM 2500 microscope.

### Non-negative matrix factorization clustering

Non-negative matrix factorization (NMF) is an effective dimensionality reduction method that is widely used to distinguish molecular patterns in high-dimensional genomic data (30, 31). Patients with GBM were divided into cluster 1 (C1) and cluster 2 (C2) based on the expression of anoikis-related genes using “NMF” R package.

### Characteristic differences of C1 and C2 subtypes

Single-sample GSEA (ssGSEA), ESTIMATE, and CIBERSORT were leveraged to quantify the TME. To further understand the differences between C1 and C2 subtypes, we investigated the difference of GBM tumor stemness index in the C1 and C2. The mutual relationships regarding the 2 potential subgroups, clinical typing of GBM, and the presence or absence of G-CIMP were demonstrated by the Sankey diagram. In addition, the differences of immune checkpoints, immune inhibitors, and immune stimulators in different subgroups of patients with GBM were also analyzed.

### One-class logistic regression

We trained a predictive model to quantify the tumor stemness using one-class logistic regression (OCLR). Briefly, we downloaded mRNA expression data from Progenitor cell biology consortium (PCBC) dataset (https://progenitorcells.org/). The transcriptomic signature was generated, and OCLR-based models are trained to evaluate the resemblance among stem cells and tumor cells. The obtained signature was used to score integrated GEO cohort using spearman correlation.

### Construction of risk score model

The differential expression genes (DEGs) between cluster1 and cluster2 were screened by differential analysis using R “limma” package. Weighted gene coexpression network analysis (WGCNA) was leveraged to identify DEGs implicated in TME, stemness, and prognosis of GBM. Then, we performed univariate COX regression analysis and least absolute shrinkage and selection operator (LASSO) regression to gain the anoikis-related genes for predicting survival and prognosis of GBM. The calculation formula of risk score is shown below:

Risk Score $\sum_{\text{j}=1}^{\text{n}}\text{Coe genej}\times \text{Exp of genej}$ The Coe (genej) was the short form of the coefficient of genes included in this study, and Exp (genej) was the expression of genes.

We systematically randomize the risk score model based on GlioVies datasets in a 7:3 ratio to distinguish the train set and the test set. We assessed the reliability of the risk score model with within-group validation through survival curves of the train set and test set. The survival rates at 1, 2, 3, and 5 years of patients with GBM were also predicted on the basis of the train set and the test set. Clinical information related to GBM survival and prognosis was obtained through the combination of risk score and clinical traits and drew clinical prognostic factors forest charts.

### Nomogram construction and characteristic of risk score model

Combining prognostic characteristics and clinical characteristics, the R package “RMS” was leveraged to perform nomogram. Calibration curves and time-dependent ROC curves were used to evaluate the risk score model as well as nomogram. We then analyzed the differences in TME and stemness between high- and low-risk groups, as well as potential associations between different subtypes of GBM.

### Pan-cancer analysis of the risk score model

We analyzed the differences between tumor mutational burden (TMB), microsatellite instability (MSI), and CD274 in 33 cancer species and clarified their upregulation and downregulation. In addition, the correlation analysis between risk score and TME as well as stemness indices were performed in pan-cancer.

### Correlation of drug sensitivity and risk score

First, we explored the percent weight of binary response and immune phenotype in high- and low-risk scores. Tumor neoantigens are antigens that are not expressed in normal tissues but only in tumor tissues, which are not only highly specific but also strongly immunogenic (32, 33). Combining risk scores and tumor neoantigens to predict prognosis of patients with GBM and plotting the survival curve. The expression information of different cell lines was obtained from Cancer Cell Line Encyclopedia. We collected drug response information and drug targeting pathways form Genomics of Drug Sensitivity in Cancer (GDSC). Then, we performed spearman correlation analysis to obtain drugs related to risk score.

### Statistical analysis

In the present experiment, all statistical analysis was conducted by R 4.1.1. The Wilcoxon test and the Kruskal–Wallis test were used for comparisons between two independent samples and comparisons among multiple samples for non-parametric data, respectively. The t-tests and one-way ANOVA were used for parametric data. P-value< 0.05 was considered statistically significant (*p-value< 0.05; **p-value< 0.01; ***p-value< 0.001). Related R packages including “ggplot2”, “ggpubr”, “survival”, and “survminer” and other related R packages were downloaded from Bioconductor packages or R packages. For each analysis, statistical significance was set at P< 0.05.

## Results

### Genetic variations and expression of anoikis-related genes in GBM

**Figure 1:**
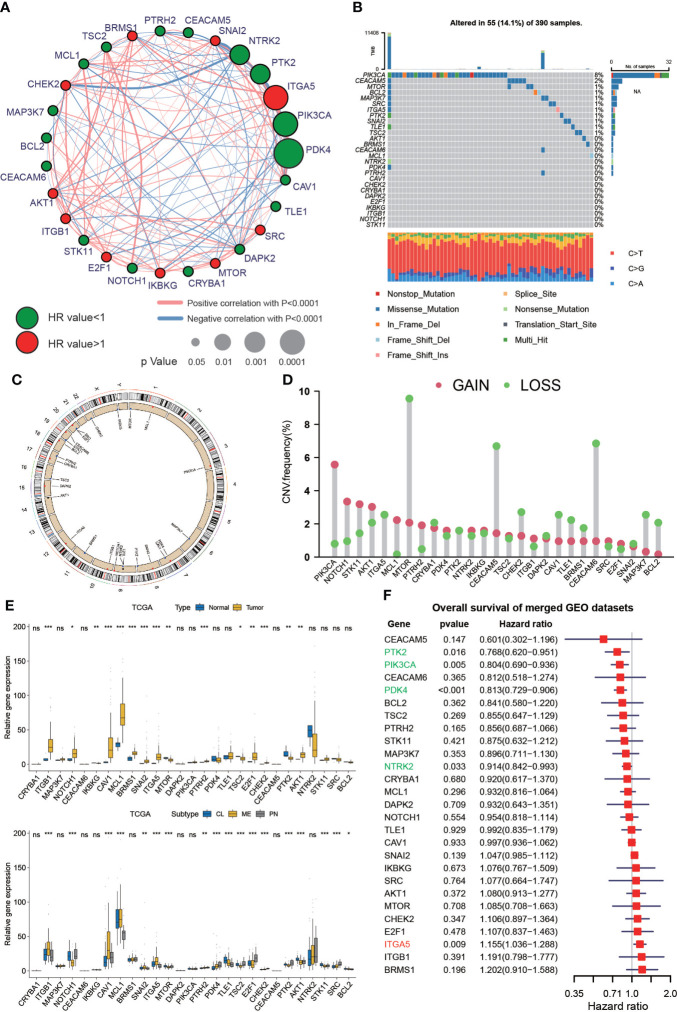
Genetic variations and expression of anoikis-related genes in GBM. **(A)** Network diagram showing the interaction of 27 anoikis-related genes in GBM. The size of the circles indicates the p-value of each gene on survival prognosis. Red represents risk factors, and green dots represent favorable factors. The thickness of the lines indicates the correlation values between genes. The red and blue lines represent positive and negative correlations of gene regulation, respectively. **(B)** Mutation prevalence of 27 anoikis-related genes in GBM. **(C)** The localization of the 27 anoikis-related genes on TCGA-GBM 23 chromosomes. **(D)** CNV variation frequency of 27 anoikis-related genes in TCGA-GBM. **(E)** Differences in expression of 27 anoikis-related genes between normal and GBM tumors and in different TCGA GBM subtypes. * means P< 0.05; ** means P< 0.01; *** means P< 0.001; ns means P > 0.05. **(F)** The effect of 27 anoikis-related genes on the overall survival of merged GEO datasets. Green represents gene downregulation and red is gene upregulation.

A total of 27 anoikis-related genes were included in this study. The comprehensive landscape of the intricate relationship between anoikis-related genes and the prognostic value for GBMs was visualized using a network plot (**Figure 1A**). We researched the somatic mutation prevalence of 27 anoikis-related genes among GBM. Among them, PIK3CA exhibited the highest mutation rate (up to 8%), whereas the mutation rates of other genes were relatively low (**Figure 1B**). CNV alterations in anoikis-related genes are visualized on chromosomes in **Figure 1C**. In addition, the investigation of 27 anoikis-related genes showed that CNV-related mutations were prevalent. PIK3CA, NOTCH1, STK11, MCL1, and PTRH2 exhibited widespread CNV amplification, whereas ITGA5, MTOR, CEACAM5, CHEK2, CAV1, TLE1, BRMS, CEACAM6, MAP3K7, and BCL2 exhibited CNV deletions (**Figure 1D**). Most anoikis-related genes were significantly upregulated in GBM tissues compared with normal tissues. In addition, we found that the expression of 27 anoikis-related genes significantly varied among CL, MES, and PN subtypes of GBM (**Figure 1E**). In our exploration of the effect of 27 anoikis-related genes on the overall survival (OS) of an integrated GEO dataset, we found that the expression of PTK2, PIK3CA, PDK4, NTRK2, and ITGA5 was statistically associated with the OS of patients with GBM (**Figure 1F**).

### Validation of PTK2 and ITGA5 expression in clinical tumor tissues

Given that the results of univariate Cox regression analysis demonstrated that PTK2 and ITGA5 were favorable and risk factors for the prognosis of patients with GBM, respectively, we further performed GSEA on PTK2 and ITGA5 to validate their gene functions. GSEA based on the merged GEO dataset showed that the APOPTOSIS, JAK STAT SIGNALING, EPITHELIAL MESENCHYMAL TRANSITION, and ANGIOGENESIS functional pathways were enriched in the ITGA5 high expression and PTK2 low expression groups, respectively (**Figures S2A, B**). Similar GSEA results were obtained on the basis of TCGA dataset (**Figures S3A, B**).

**Figure 2:**
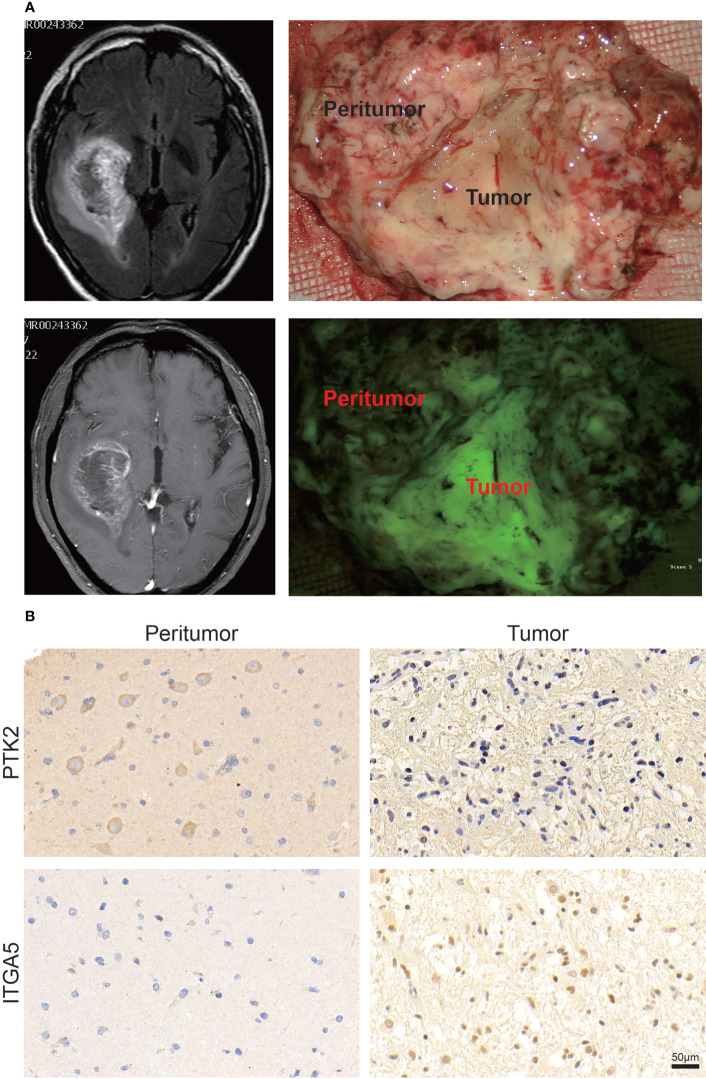
Validation of PTK2 and ITGA5 expression in clinical tumor tissues. **(A)** Representative images of specific clinical specimens of tumor and peritumor tissue. Tumor fluorescence was used to preliminarily identify the tumor. **(B)** Representative images of IHC staining for PTK2 and ITGA5 in tumor and peritumor tissue (scale bar: 50 μm).

Immunohistochemistry (IHC) staining was performed to detect the representative PTK2 and ITGA5 protein levels in gliomas and peritumor tissues (16 cases) obtained from patients treated at The Second Hospital of Shandong University. Consistent with the previous results, ITGA5 protein levels were significantly increased in glioma compared to peritumor tissue, whereas PTK2 levels were decreased in tumors (**Figures 2A, B**).

### Correlation of anoikis pattern with TME, stemness, and clinical traits

**Figure 3:**
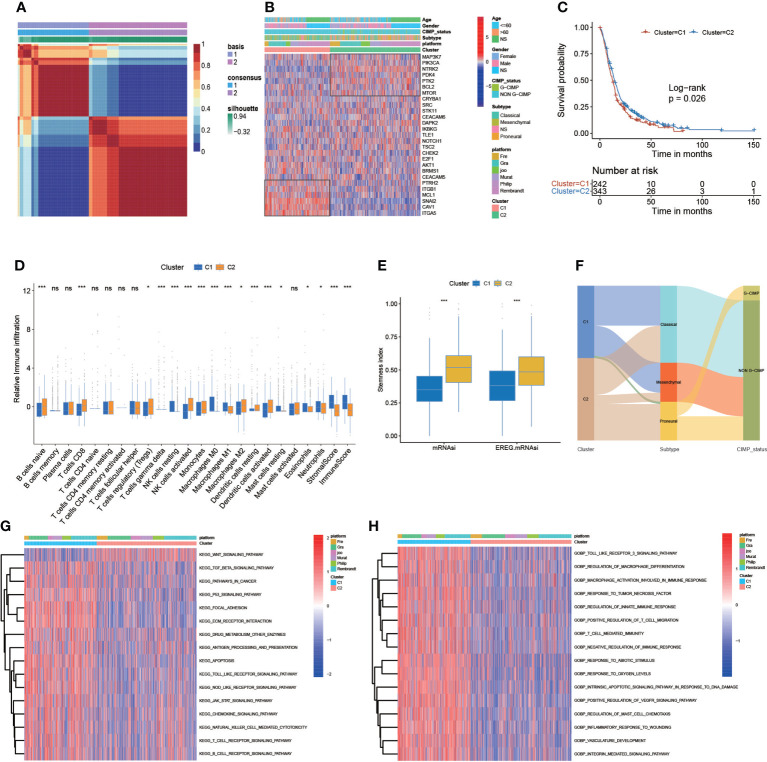
Correlation of anoikis pattern with TME, stemness, and clinical traits. **(A)** The patients with GBM were divided into two distinct gene clusters (C1 and C2) by using non-negative matrix factorization (NMF). **(B)** The sample annotation of 27 anoikis-related genes in gender, age, platform, IDH status, CIMP_status, and cluster. **(C)** Survival analyses for the C1 (242 cases) and C2 (343 cases) cohorts (p = 0.026, log-rank test). **(D)** The abundance of each TME-infiltrating cell, stromal scores, and immune scores in C1 and C2 clusters. **(E)** The stemness index difference of the two gene clusters. **(F)** The Sankey diagram is about the relationship between C1 and C2 clusters, clinical typing, and cytosine-phosphate-guanine (CpG) island methylator phenotype (G-CIMP). **(G, H)** KEGG analyses and GO analyses for anoikis-related genes of the two gene clusters. (*p-value < 0.05; **p-value < 0.01; ***p-value < 0.001).

We used the NMF algorithm to classify 650 patients with GBM into two clusters, termed C1 and C2 (**Figure 3A**). The heatmap visualized the detailed expression of anoikis-related genes in the C1 and C2 clusters (**Figure 3B**). The result of the Kaplan–Meier (KM) analysis showed the difference in patient survival between the C1 and C2 clusters (p = 0.026, log-rank) (**Figure 3C**). Regarding TME differences between the two clusters, we observed that C1 was remarkably abundant in several immune cell infiltrates, such as regulatory T cells, CD8 T cells, activated NK cells, dendritic cells, and others (**Figure 3D**). In addition, the regulatory role of anoikis-related genes on immune-related cell expression in GBM tumors was analyzed and is displayed in **Figure S4A**. In the stemness index analysis, the mRNAsi and EREG-mRNAsi of C2 were much closer to 1, suggesting that GBM in C2 has a high similarity to stem cells (**Figure 3E**). However, some studies have shown that higher indices seem to be directly related to the degree of progression of many types of cancer and poor prognosis, so further research is needed. In the Sankey diagram, we identified the interrelationship between the C1 and C2 subtypes and clinical typing, and the patients with GBM with the cytosine-phosphate-guanine (CpG) island methylator phenotype (G-CIMP) were mainly concentrated in C2 (**Figure 3F**). We assessed the TME in GBM tumor tissues. Chemokines, immune inhibitors, and immune stimulators were more significantly expressed in C1 (**Figure S3B**). Kyoto Encyclopedia of Genes and Genomes (KEGG) analysis revealed that the following pathways were significantly activated in C2: cancer, apoptosis, the T-cell receptor signaling pathway, the B-cell receptor signaling pathway, and others. Interestingly, the Wnt signaling pathway was much more active in C1 (**Figure 3G**). In addition, Gene Ontology (GO) biopathway analysis revealed that anoikis-related genes in C1 were significantly enriched in the following functional sets: toll-like receptor 3 signaling pathway, response to tumor necrosis factor, T-cell–mediated immunity, and others (**Figure 3H**).

### Construction and validation of the risk score

**Figure 4:**
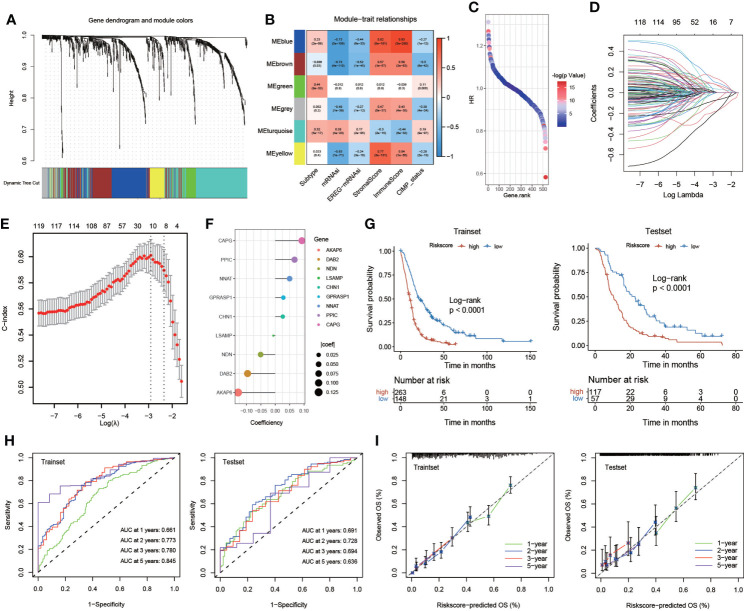
Construction and validation of the risk score. **(A)** Weighted gene coexpression network analysis (WGCNA) based on gene expression data identified gene modules with highly synergistic changes. **(B)** The heatmap of module-trait relationships. **(C)** Univariate Cox regression analysis of 524 genes relevant to GBM prognosis. **(D, E)** The least absolute shrinkage and selection operator (LASSO) method of anoikis-related genes associated with prognosis. **(F)** The risk score for predicting the survival and prognosis of patients with GBM. **(G)** Kaplan–Meier curves of the train set (p< 0.001, log-rank test) and test set (p< 0.001, log-rank test). **(H)** Time-dependent receiver operating characteristics (ROC) of the train set and the test set. **(I)** Calibration curves for risk score model in the train set and the test set.

To obtain the key module most implicated in clinical characteristics, we performed WGCNA on the merged GBM datasets (**Figure 4A**). We obtained a total of 897 differential genes associated with anoikis, and coexpression modules were eventually identified. According to the heatmap of module-trait relationships, the MEblue and MEturquoise modules demonstrated the highest correlations with clinical traits (**Figure 4B**). A univariate Cox regression algorithm was performed to preliminarily acquire 524 genes relevant to GBM prognosis and calculate the HR and P-values for the anoikis-related genes. The results are shown in **Figure 4C**. Next, we sought to identify prognostic gene sets for GBM using the LASSO algorithm and ultimately found nine gene sets (**Figures 4D, E**). Finally, nine anoikis-related genes for predicting the survival and prognosis of patients with GBM were used to construct the risk score (**Figure 4F**).

We systematically randomized the cohort of GlioVies-GBM patients to distinguish the training set (n = 411) and the test set (n = 174). KM analyses revealed that a higher risk score in the training set and the test set corresponded with poorer survival (P< 0.0001) (**Figure 4G**). Time-dependent receiver operating characteristics (ROCs) and decision curve analysis (DCA) were utilized to assess the sensitivity and specificity of the model for prognosis. The outcomes were assessed on the basis of the area under the ROC curve (AUC). The 1-, 2-, 3-, and 5-year AUCs of the training set were 0.661, 0.773, 0.780, and 0.845, respectively, and those of the test set were 0.691, 0.728, 0.694, and 0.636, respectively (**Figure 4H**). We also validated the accuracy of the ROC to predict the prognosis of patients with GBM (**Figure 4I**). Multivariate Cox regression analyses were used to assess whether clinical characteristics (gender, CIMP status, and subtype) and risk score were independent prognostic factors for patients with GBM. We found that age and risk score were independent prognostic factors for patients with GBM in the training set and the test set (**Figure S4D**).

### The nomogram based on risk score in GBM

**Figure 5:**
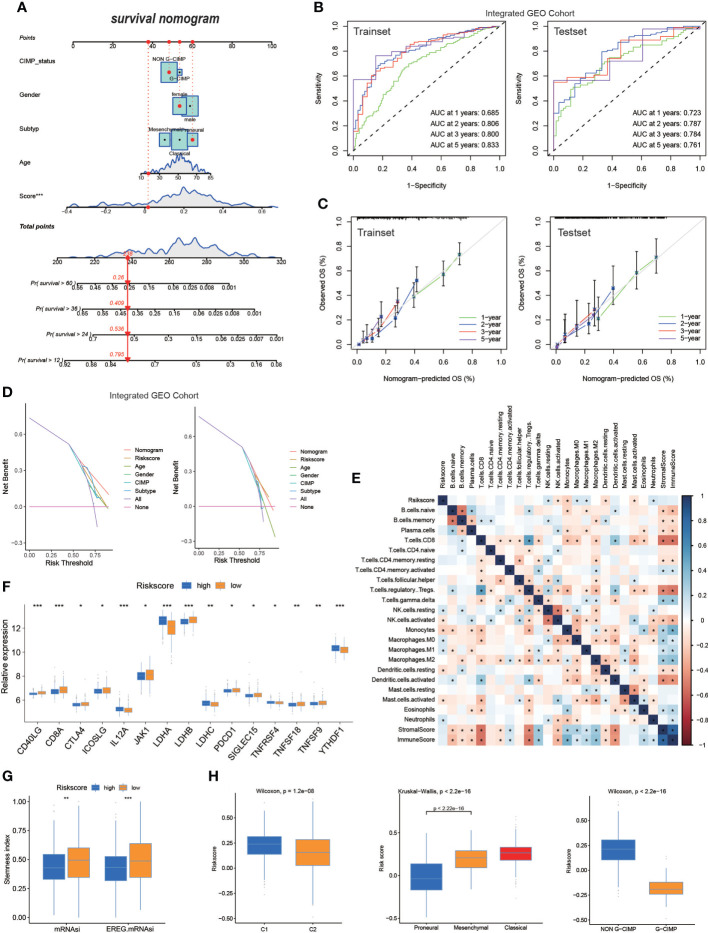
The nomogram based on risk score in GBM. **(A)** Nomogram integrated the age, gender, CIMP_status, subtype, and risk score. **(B)** The ROC of the train set and the test set containing nomogram. **(C)** Validation of the ROC of the train set and the test set containing nomogram. **(D)** The decision curve analysis **(DCA)** of the train set and the test set containing nomogram. **(E)** The correlation of risk score and TME infiltration cells. **(F)** Expression of immune checkpoints among high– and low–GBM risk groups. **(G)** The stemness index difference of high- and low-risk score groups. **(H)** The relationship between the risk score model and two clusters, GBM subtypes, and G-CIMP subtypes. (*p-value < 0.05; **p-value < 0.01; ***p-value < 0.001).

The hybrid nomogram was stable and accurate and may be applied in the clinical management of patients with GBM. We built a nomogram capable of predicting the survival probabilities of GBM at 1, 2, 3, and 5 years based on the high- and low-risk score models (**Figure 5A**). The nomogram was also incorporated into time-dependent ROCs to predict the survival time of patients with GBM. The 1-, 2-, 3-, and 5-year AUCs of the training set were 0.685, 0.806, 0.800, and 0.833, respectively, and those of the test set were 0.723, 0.787, 0.784, and 0.761, respectively (**Figure 5B**). Validation and evaluation of the ROC are shown in **Figure 5C**, and the DCAs of the risk factors are displayed in **Figure 5D**.

Regarding differences in TME infiltration between high and low risk scores, we observed that several immune-related factors in the lower risk score group were remarkably abundant (**Figure 5E**). Regarding the immune checkpoint, CD40LG, CD8A, JKA1, LDHB, and others were more pronouncedly highly expressed in patients with lower risk scores, and IL12A, LDHA, LDHC, TNFRSF4, and YTHDF1 were significantly expressed in patients with higher risk score (**Figure 5F**). The mRNAsi and EREG-mRNAsi in patients with a lower risk score were much closer to 1, suggesting that that patients with GBM with lower risk score were less differentiated compared with patients with GBM with higher risk score (**Figure 5G**); this finding requires more research. When analyzing the relationship between the risk score model and different subgroups of patients with GBM, the Wilcoxon test revealed an obvious difference in the risk score between C1 and C2. Moreover, C2 showed a lower median risk score than C1. In addition, we found an obvious difference in the risk score between the G-CIMP and non-G-CIMP groups and between the PN and MES GBM subtypes (**Figure 5H**).

### Validation of the risk score based on TCGA dataset

**Figure 6:**
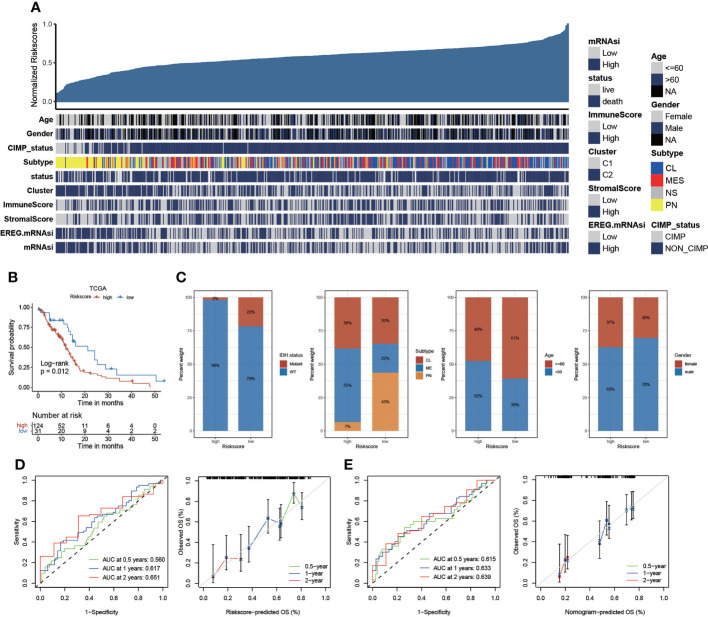
The risk score based on TCGA datasets. **(A)** The relationship between risk scores and clinical characteristics. **(B)** Kaplan–Meier curves of the risk score based on TCGA datasets (p = 0.012, log-rank test). **(C)** The proportion of clinical characteristics in high- and low-risk scores. **(D)** The AUC of the 1-, 2-, and 3-year survival rate of GBM and validation of the ROC of the risk score. **(E)** The AUC and validation of the ROC of the nomogram model.

We have constructed risk score based on integrated GEO cohort and analyzed the correlation between risk score and clinical characteristics (**Figure 6A**). We further validated the risk scoring model based on the same algorithm generated using samples obtained from TCGA datasets. We validated the risk score based on differentially expressed anoikis-related genes based on survival analysis, and we also found that KM analyses revealed that the expression of high-risk genes corresponded with poorer survival (**Figure 6B**). The percent weight of different clinical prognostic factors in patients with GBM of the high- and low-risk groups is shown in **Figure 6C**. In addition, we compare the risk score between men and women, and the results exhibited that there was no significant difference of risk score between men and women (**Figure S5B**). However, patients with GBM aged >60 years had significantly higher risk scores than those aged<60 years (**Figure S5C**). We illustrated the outcomes of ROC with the AUC. The 0.5-, 1-, and 2-year AUCs of the risk scores were 0.560, 0.617, and 0.651, respectively, exhibiting superior performance than the traditional clinicopathological features in predicting the prognosis of GBM. We also validated the accuracy of the ROC based on the risk score to predict the prognosis of patients with GBM (**Figure 6D**). Then, the AUC and the accuracy of the ROC, for which we developed a nomogram based on the risk score, were analyzed (**Figure 6E**). Furthermore, we evaluated the tumor somatic mutations presented in high- and low-risk patients separately using the map tools package. The results showed that the low score group presented a more extensive TMB than the high score group (**Figure S5A**). In addition, somatic mutations in several genes, such as TP53, are rarely observed in the low-risk group but are frequently observed in the high-risk group. TP53, which regulates cell division and proliferation, is a tumor suppressor gene. Many studies have shown that mutations in the TP53 gene are associated with the development of a variety of human tumors (34, 35). We further validated the risk scoring model based on CGGA cohort. The outcomes were similar to the previous analysis. Higher risk score group patients with GBM exhibited shorter survival time (**Figure S5D**). In addition, the time–ROC and calibration curves for CGGA GBM were also visualized in **Figures S5E, F**.

### The pan-cancer analysis of risk score model

**Figure 7:**
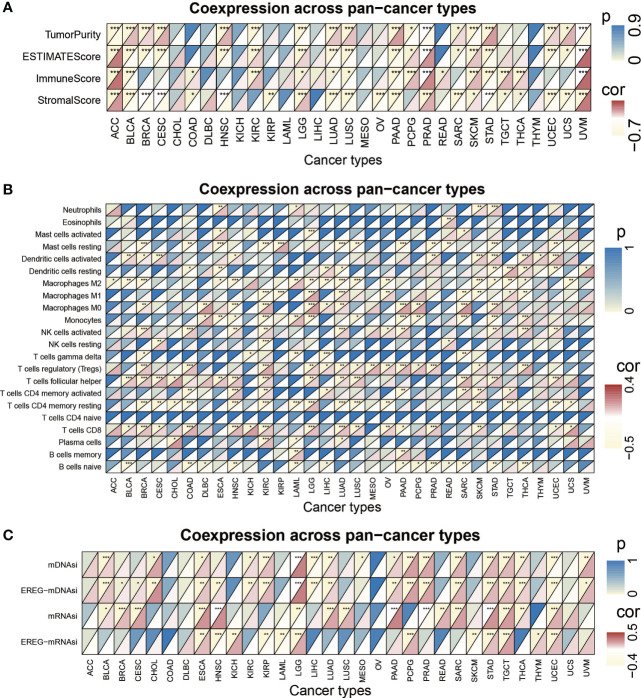
The pan-cancer analysis of risk score model. **(A)** Tumor purity, ESTIMATES score, immune score, and the stromal score of 32 types of tumors. **(B)** The TME-infiltrating cell of 32 types of tumors. **(C)** The stemness index difference of 32 types of tumors. (*p-value < 0.05; **p-value < 0.01; ***p-value < 0.001).

We performed pancancer analysis to evaluate the similarity and difference of the risk score model between different cancers (**Figure S6A**) (36). We systematically analyzed TMB (37, 38), MSI (39, 40), and the expression of CD274 across cancers (41–43). The risk score was positively correlated with TMB in BRCA, CESC, COAB, ESCA, HNSC, LGG, LUAD, PRAD, STAD, and THCA (P< 0.05) but negatively correlated with TMB in KIRC and LAML (P< 0.05). For MSI, a positive association in COAD, DLBC, ESCA, HNSC, SARC, STAD, UCEC, and THCA, as well as a negative association in TGCT, was identified. In addition, the risk score was positively correlated with CD274 expression in ACC, LGG, PCPG, PRAD, SKCM, TGCT, and THCA and negatively associated with CD274 content in BRCA, ESCA, KIRP, LAML, LUAD, and PAAD (**Figure S6B**). In addition, we calculated the correlation between the risk score and 22 immune cell infiltration and stemness indices. The results are displayed in **Figures 7A–C**.

### Correlation of risk score with immunotherapy response and drug sensitivity

**Figure 8:**
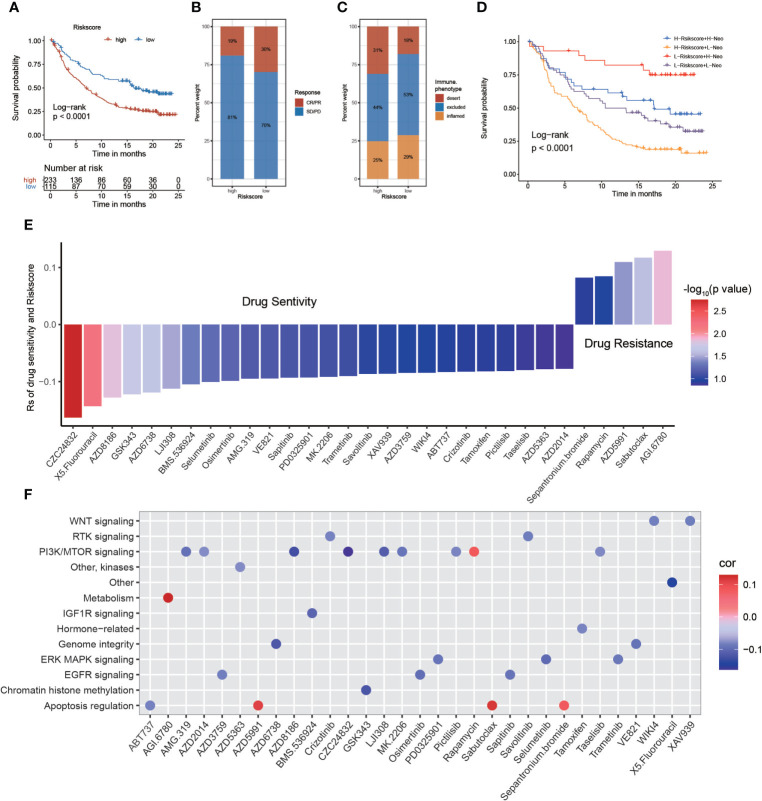
The relationship between risk score and response to immunotherapy and drug sensitivity. **(A)** Survival analysis of the low and high-risk score patient groups in an immune checkpoint blockade (ICB) therapy cohort. **(B)** The immunotherapy response and immune phenotypes of patients with GBM in high- and low-risk scores. **(C, D)** Survival analyses of patients with GBM receiving ICB therapy by risk score combining tumor neoantigen burden. **(E)** Assessing drug sensitivity of GBM tumor based on the risk score. **(F)** Signal paths targeted by drug sensitivity with the risk score. Blue (positive correlation) or red (negative correlation).

To evaluate the effect of the risk score on predicting the sensitivity of immunotherapy, we included an immunotherapy cohort of advanced urothelial cancer (IMvigor210 cohort). The log-rank test shows that patients with higher risk scores are associated with poorer survival time (**Figure 8A**). Immune checkpoint blockade (ICB) works by blocking the interaction between tumor cells expressing immune checkpoints and immune cells, thereby relieving the suppressive effect of tumor cells on immune cells (44–46). We evaluated the value of the risk score in predicting the response to immunotherapy. We observed that patients who had lower risk scores exhibited better treatment results in the ICB therapy research and concluded that the proportion of patients in the response groups (CR and PR) was significantly lower in the high-risk score group compared with the low-risk score group. However, the proportion of patients in the no/limited response groups (SD and PD) showed the opposite trend, indicating that the risk score could reveal the response of patients to ICB therapy (**Figure 8B**). Regarding the relationship between high- and low-risk groups and immune phenotypes, the desert phenomenon was more pronounced in the high-risk group, whereas more inflammation was observed in the low-risk group (**Figure 8C**). By analyzing the relationship between the risk score with tumor neoantigen burden, we observed that patients with lower risk score together with a high neoantigen burden exhibited the most prolonged survival time. In addition, patients with higher risk score and a low neoantigen burden had the shortest survival time (**Figure 8D**).

The value of the risk score to predict drug sensitivity in multiple cancer types was evaluated. According to Spearman correlation analysis, we selected 31 drugs for which the risk score and drug sensitivity were significantly correlated in the GDSC database. The risk score was negatively sensitive to five drugs, including sepantronium bromide, rapamycin, AZD5991, sabutoclax, and AGI-6780, and positively correlated with sensitivity to 26 drugs, including CZC24832, 5-fluorouracil, AZD8186, GSK343, and others (**Figure 8E**). Among them, CZC24832 exhibited the strongest drug sensitivity. The study showed that pharmaceutical PI3Kγ inhibition with CZC24832 significantly impaired CLL cell migration (47). Furthermore, we explored the signaling pathways targeted by the selected drugs. We uncovered that the relationship between drug sensitivity and risk scores based on PI3K/mTOR, IGF1R, genome integrity, and EGFR signaling was positive. In contrast, drugs with a sensitivity that was negatively related to the risk score targeted the metabolism and apoptosis regulation signaling pathways (**Figure 8F**). In conclusion, the establishment of the risk score will facilitate the exploration of the correct and effective treatment strategy.

## Discussion

Anoikis is an important defense of the organism, preventing the readhesion of shed cells to a new substrate in an incorrect location and preventing their stunted growth (9). Some studies have demonstrated that the occurrence of anoikis apoptosis depends on the intrinsic pathway and the extrinsic pathway (48). Anoikis can be triggered in response to several intracellular signals, including DNA damage and endoplasmic reticulum stress, whereas mitochondria play a central role in controlling apoptosis (49). This disorder in the execution of anoikis potentially represents a hallmark of cancer cells and contributes tumor invasion and migration, the formation of distant organ metastasis and the development of drug resistance (50–52). However, there are few studies on the effects of anoikis-related genes on invasive mobility and drug resistance in GBM and their role in predicting the prognosis of GBM.

In our research, we provide a full view of the differential expression of hallmark gene sets in GBM between tumor tissues and normal tissues and the effect on altered immune activity. The unsupervised consistency clustering algorithm was used to classify patients with GBM, and we eventually obtained two potential subgroups. We analyzed and evaluated the potential subgroups, and the results showed a difference in the survival time of patients with GBM between the two subgroups. In addition, immune cell infiltration and immune targets were analyzed to identify differences between subgroups. Next, we constructed risk models based on differences in gene expression and predicted patient survival and prognosis based on the risk models. A risk score model was constructed according to gene expression to analyze and verify the role of the risk scoring model in predicting the prognosis of patients with GBM. In addition, the possibility of immunotherapy in GBM was discussed on the basis of the risk scoring model. Currently, there is increasing evidence that the presence of anti-anoikis-related genes is closely related to the ability of tumors to migrate aggressively and drug resistance in a variety of tumors. A study showed that androgen-dependent prostate cancer cells become resistant due to roadblocks in anoikis and, depending on interactions with the tumor microenvironment, acquire invasive and metastatic properties (53). The emergence of both anti-apoptotic and pro-metastatic signaling mechanisms was also identified in LKB1-deficient lung cancer. The mechanism involves the enhancement of its substrate AMPK binding by the GDH1 product α-KG that activates CamKK2 to generate energy, resulting in anti-anoikis and ultimately promoting metastasis of lung cancer (54). Here, we revealed global anoikis-related gene alterations at the genetic and transcriptional levels and showed mutual correlations in GBMs. Interestingly, the anoikis-related genes interacted and influenced each other. Specifically, PDK4 gene downregulation exhibited a positive effect on patient survival, and ITGA5 gene upregulation yielded a negative effect on the survival of patients with GBM. We constructed a risk score model to predict patient prognosis and the response to immunotherapy and targeted therapy. Exploring the differential expression of anoikis-associated genes in GBM not only improves our knowledge of the aggressiveness of GBM but also contributes to the development of more personalized and precise immunotherapy regimens.

Evidence is now available to demonstrate that immunotherapy can benefit patients with GBM; however, the lack of understanding of the tumor microenvironment and immune cell infiltration in GBM has resulted in patients receiving immunotherapy without obtaining effective results (55–57). In addition, many immunotherapy strategies that have yielded successful results in preclinical studies have failed to produce convincing results in clinical trials, revealing the limitations and inadequacies of current preclinical models of GBM (58–60). Therefore, we constructed a risk score model for GBM prognosis based on the difference in anoikis-related gene expression in this experiment. We examined the value of the risk score in predicting the response of GBM to immunotherapy and analyzed the differences in the expression of immune-related cells tumors with high and low risk scores. We also observed that patients who had lower risk scores exhibited obviously prolonged OS in ICB therapy research. This finding demonstrates the reliability of using this risk score model to predict the efficacy of immunotherapy in patients. When investigating the treatment outcome based on the risk score in patients with GBM, we found an interactive relationship between drug sensitivity and risk score. Apoptosis regulation signaling and metabolism play an active role in the treatment of GBM. In contrast, drugs with sensitivity that was negatively related to the risk score targeted PI3K/mTOR, IGF1R, genome integrity, and the EGFR signaling pathway. These findings indicated that patients with higher risk scores might be more suitable for drugs targeting metabolism and apoptosis regulation signaling instead of the PI3K/mTOR, IGF1R, genome integrity, and EGFR signaling pathways.

## Conclusion

In this research, we systematically generated and assessed the risk score of GBMs based on 27 anoikis-related genes and linked these patterns with the TME. The risk score model was utilized to predict patient prognosis and response to immunotherapy. The systematic assessment of the risk score could expand our understanding of invasion and contribute to the development of more personalized and precise therapy strategies.

## Data availability statement

The original contributions presented in the study are included in the article/**Supplementary Material**. Further inquiries can be directed to the corresponding author.

## Ethics statement

The studies involving human participants were reviewed and approved by Ethics Committee of The Second Hospital of Shandong University. The patients/participants provided their written informed consent to participate in this study. Written informed consent was obtained from the individual(s) for the publication of any potentially identifiable images or data included in this article.

## Author contributions

CW led study design and prepared the manuscript. ZS, CT performed the research and wrote the manuscript. YZ, YW and XD collected clinical samples and corresponding clinical data. CW revised the manuscript. All authors contributed to the article and approved the submitted version.

## Funding

This work was supported by grants from the National Natural Science Foundation of China (No.82103144), Key Research and Development Program of Shandong Province (Major Scientific and Technological Innovation Project) (No. 2021CXGC011101), and China Postdoctoral Science Foundation (No. 2021M691942).

## Conflict of interest

The authors declare that the research was conducted in the absence of any commercial or financial relationships that could be construed as a potential conflict of interest.

## Publisher’s note

All claims expressed in this article are solely those of the authors and do not necessarily represent those of their affiliated organizations, or those of the publisher, the editors and the reviewers. Any product that may be evaluated in this article, or claim that may be made by its manufacturer, is not guaranteed or endorsed by the publisher.
