# Supplementary material for: Identification and validation of an anoikis-associated gene signature to predict clinical character, stemness, IDH mutation, and immune filtration in glioblastoma
Source: Front Immunol. 2022 Aug 25;13:939523. doi: 10.3389/fimmu.2022.939523 (PMC9452727; doi:10.3389/fimmu.2022.939523)
Supplement: Supplementary file 1 [file DataSheet_1.docx]

**Supplementary Figures**

**
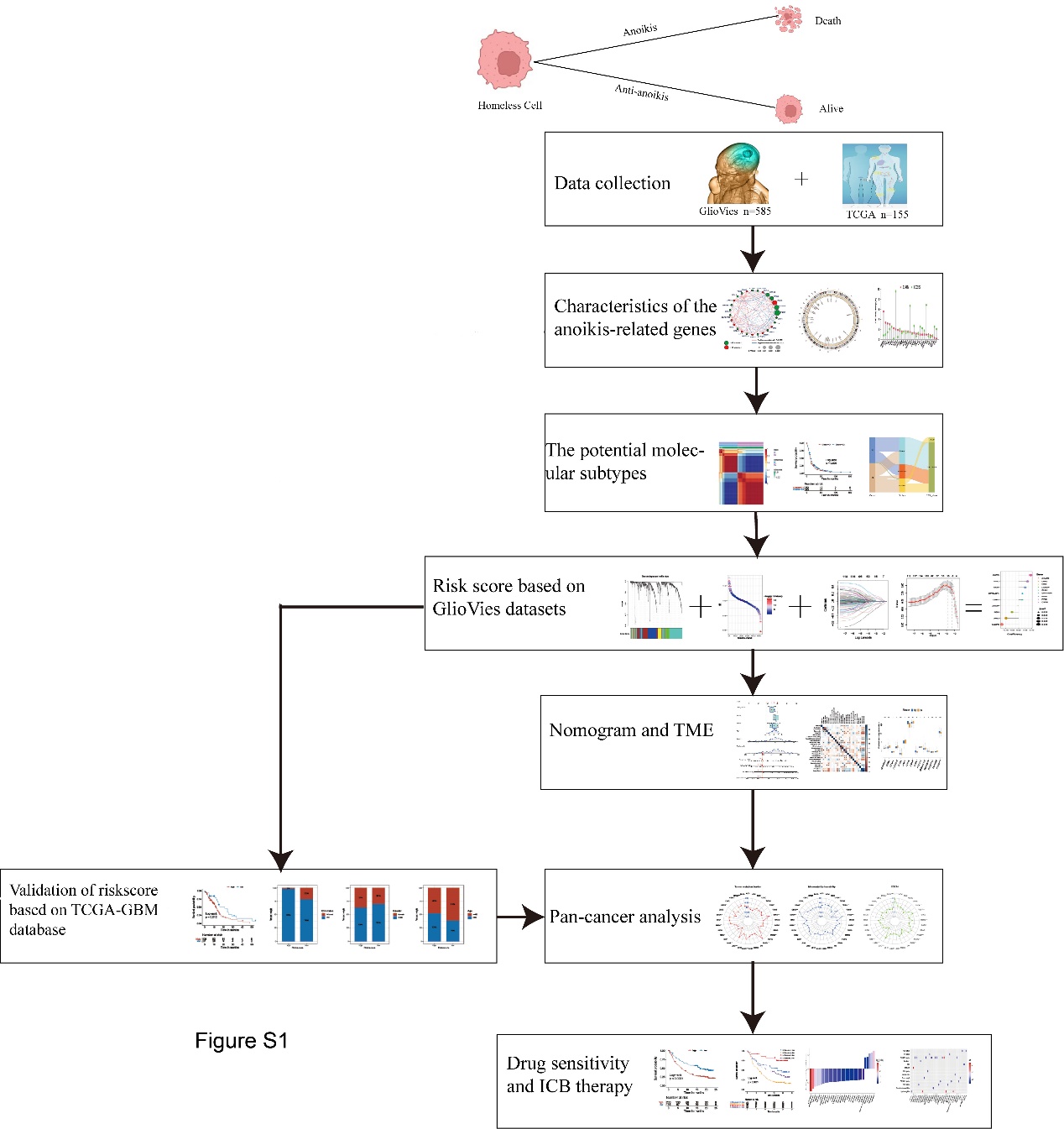
**

**Figure S1** Landscape of this study workflow.

**
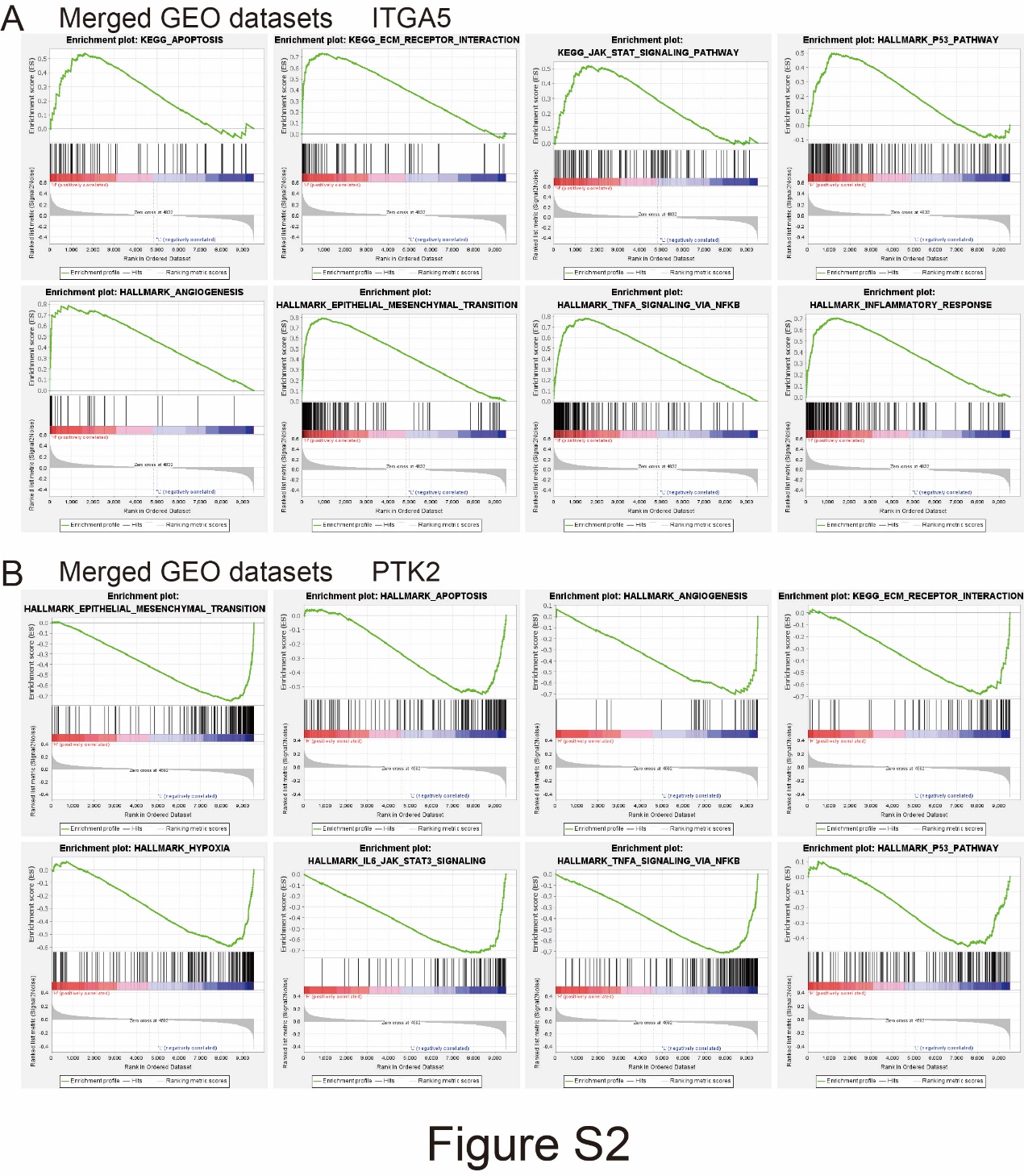
**

**Figure S2 A B** Gene Set Enrichment Analysis for ITGA5 (**A**) and PTK2 (**B**) based on merged GEO datasets.

**
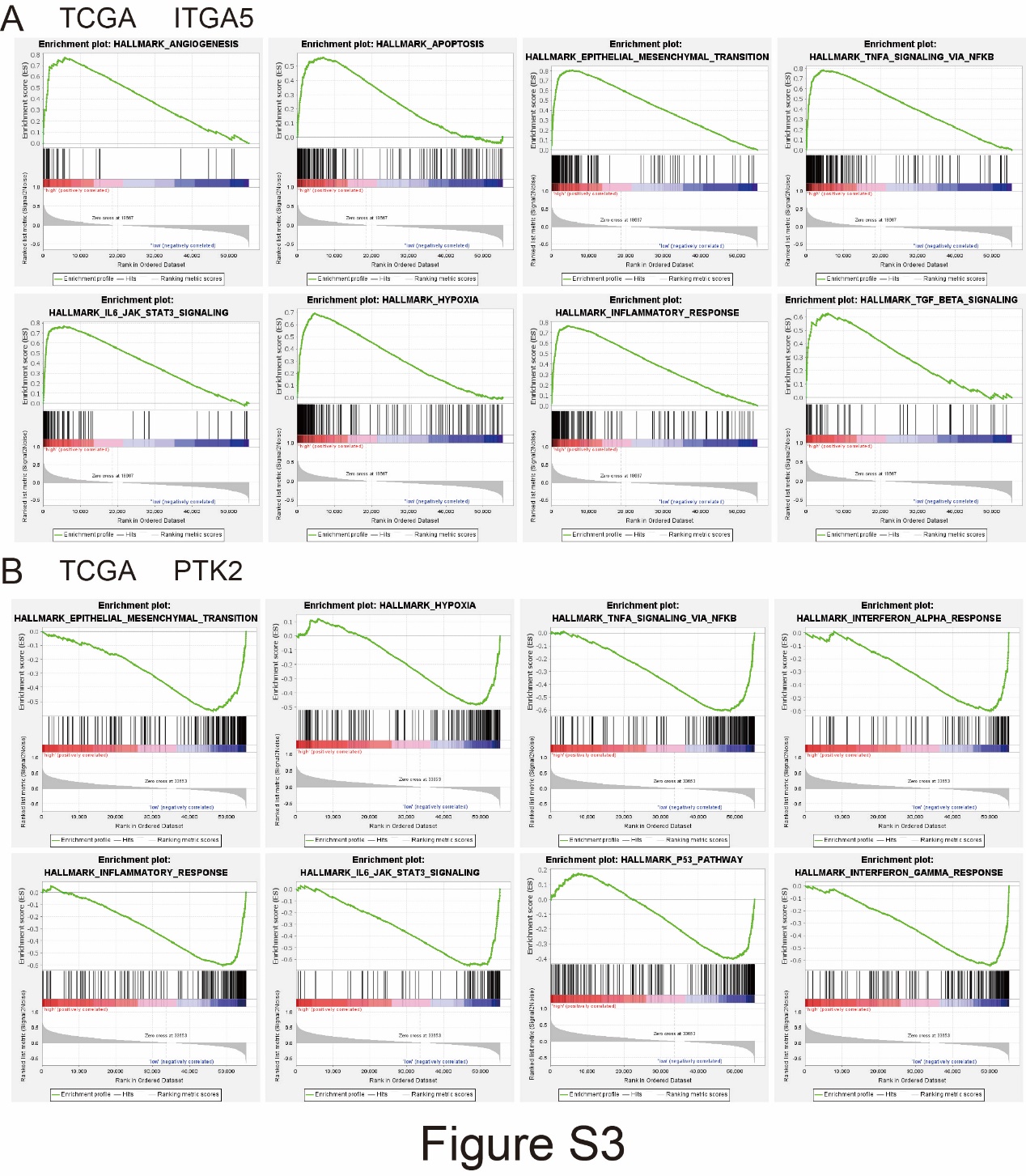
**

**Figure S3 A B** Gene Set Enrichment Analysis for ITGA5 (**A**) and PTK2 (**B**) based on TCGA dataset.

**
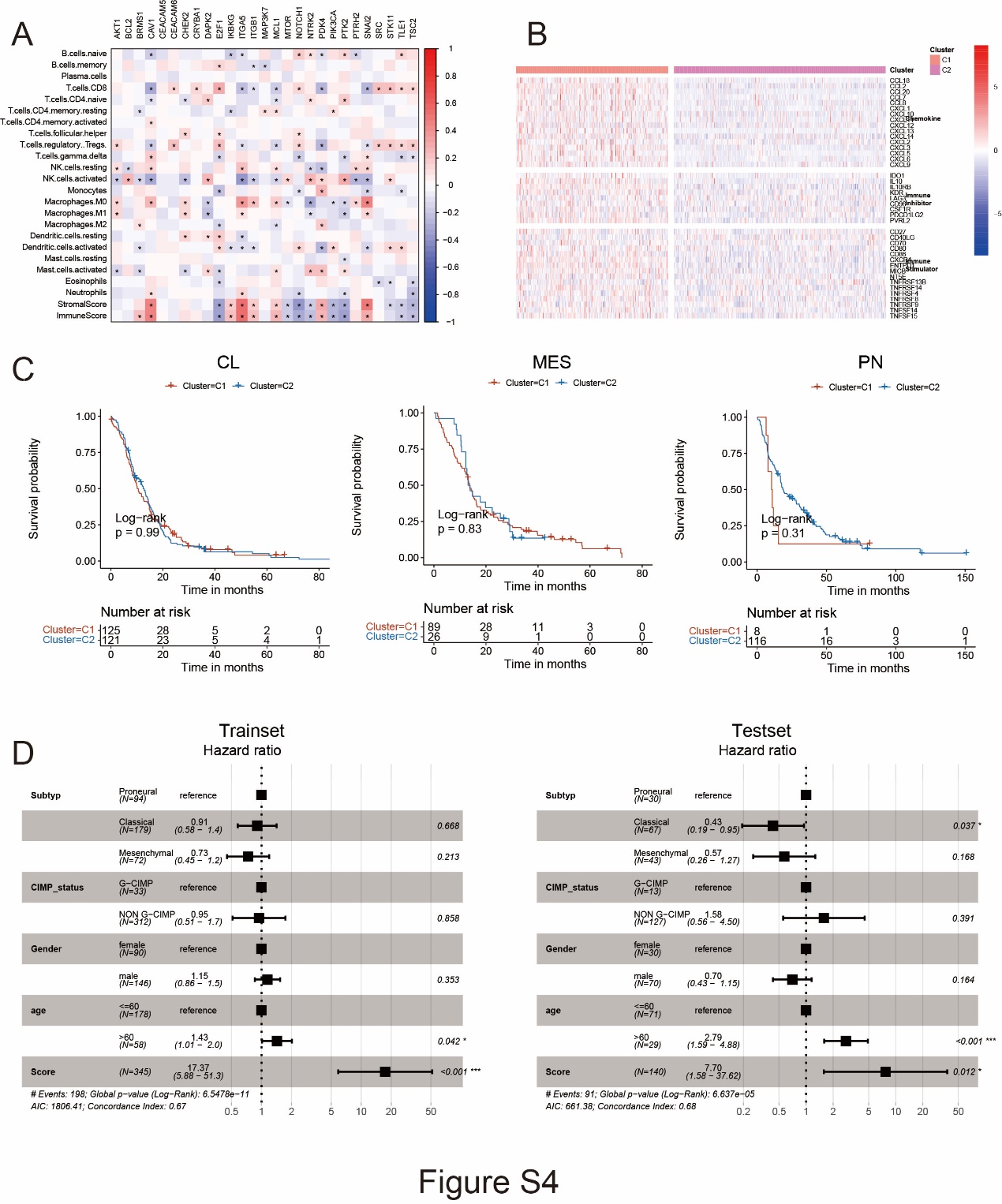
**

**Figure S4 A** The correlation of anoikis-related genes and immune-related cell expression in GBM tumors. **B** The differences of Chemokine, immune inhibitor, and immune stimulator between C1 and C2 clusters. **C** The subgroup survival analysis and compared the prognosis of C1 and C2 in each GBM subtype separately. **D** Assessing clinical independent prognostic factors for GBM Patients in Trainset and Testset, respectively.

**
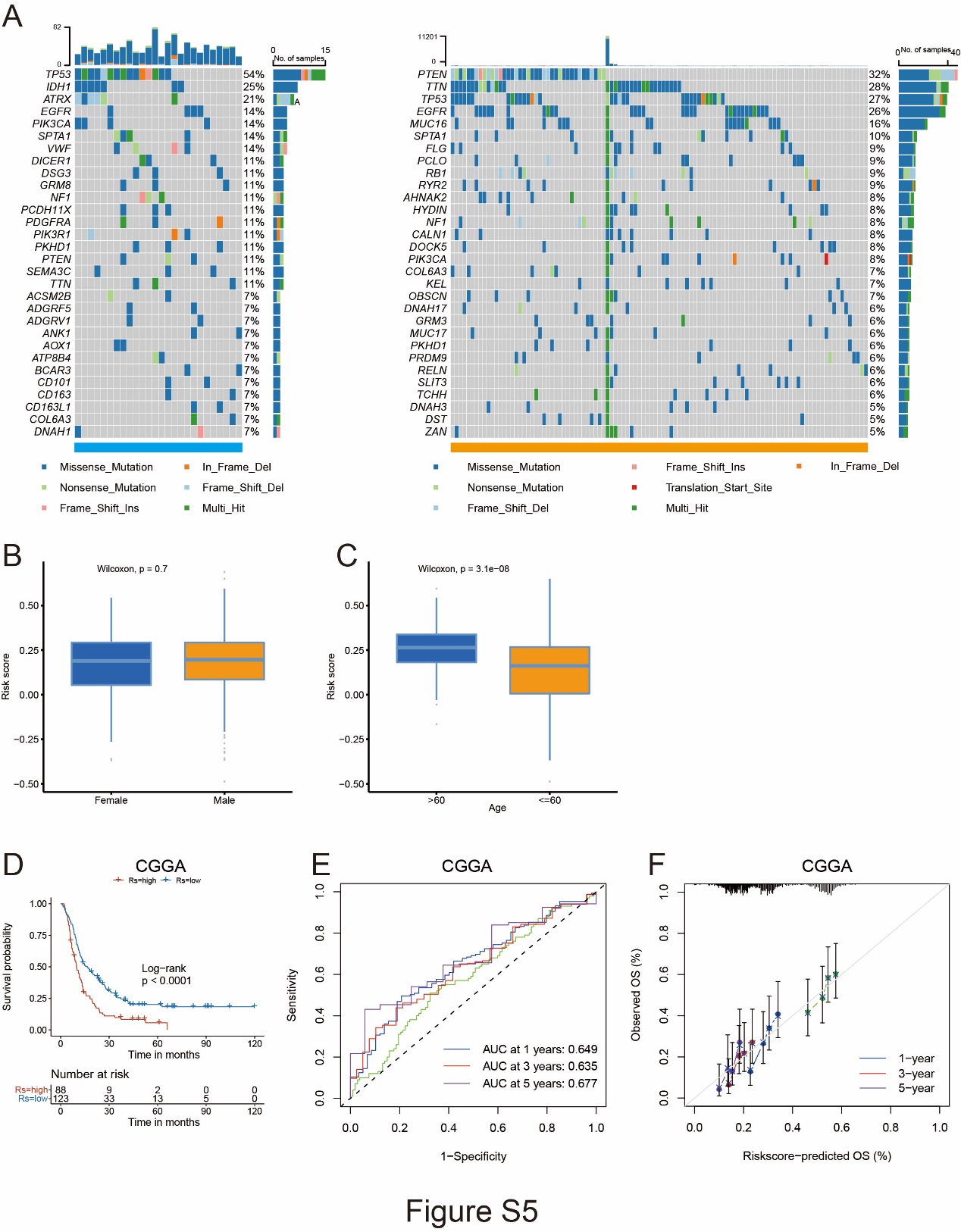
**

**Figure S5 A** Waterfall plot showing tumor mutational burden (TMB) presented by those with high-risk score and low-risk score group. **B** The difference of risk score between males and females. **C** The difference of risk score between GBM patients aged >60 years and those aged <60 years.

**
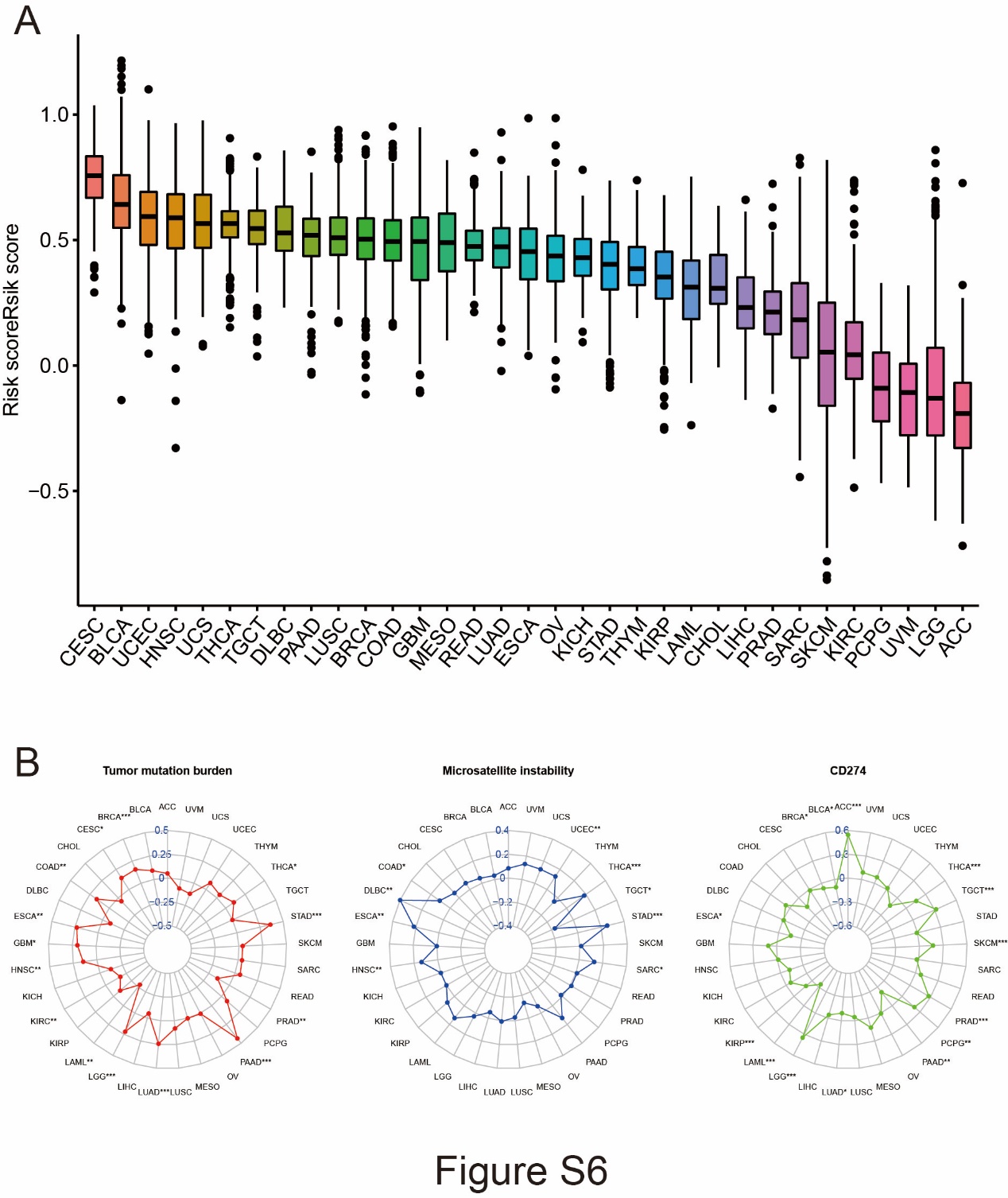
**

**Figure S6 A** The risk score distribution in pan-cancer. **B** Tumor mutation burden (TMB), microsatellite instability (MSI) and CD274 of pan-cancer.
